# Evaluating the antibacterial properties of deep-sea sponges *Dactylospongia elegants*, *Stelletta fibrosa*, and *Haliclona manglaris* from the Jordanian Gulf of Aqaba

**DOI:** 10.7717/peerj.19735

**Published:** 2025-07-31

**Authors:** Razan Ataallah Abuassaf, Fatima F. Al-Jamal, Osama H. Abusara, Malek Zihlif, Ahmad A. Deeb, Mamoon M.D. Al-Rshaidat

**Affiliations:** 1 Department of Biological Sciences, School of Sciences, The University of Jordan, Amman, Jordan; 2 Department of Chemistry, Faculty of Science, Isra University, Amman, Jordan; 3 Laboratory for Molecular and Microbial Ecology (LaMME), The University of Jordan, Amman, Jordan; 4 Department of Biological Sciences, Faculty of Science, Jerash Private University, Jerash, Jordan; 5 Department of Pharmacy, Faculty of Pharmacy, Al-Zaytoonah University of Jordan, Amman, Jordan; 6 Department of Pharmacology, School of Medicine, The University of Jordan, Amman, Jordan

**Keywords:** Deep marine sponges, Gulf of Aqaba, Bioactive compounds, Antimicrobial resistance (AMR), Marine natural products, DNA barcoding, Antibacterial activity

## Abstract

Marine sponges are known for their rich variety of secondary metabolites, many of which show potential for pharmaceutical applications. In this study, three deep-sea sponge species—*Stelletta fibrosa*, *Dactylospongia elegans,* and *Haliclona manglaris*—were identified using DNA barcoding, and their ethanolic extracts were tested for antibacterial activity. The extracts were evaluated against Gram-positive (*e.g.*, *Bacillus pumilus, Staphylococcus aureus, Staphylococcus epidermidis,* and methicillin-resistant *Staphylococcus aureus*, MRSA) and Gram-negative bacteria (*e.g.*, *Escherichia coli* and *Klebsiella aerogenes)* using the agar well diffusion method. The minimum inhibitory concentration (MIC) and minimum bactericidal concentration (MBC) were also determined. Among the extracts, *D. elegans* exhibited the most potent antibacterial activity, with inhibition zones ranging from six to 21 mm against gram-positive bacteria and low MIC/MBC values from 0.25 to three mg/ml. Liquid chromatography-mass spectrometry (LC-MS/MS) analysis of *D. elegans* revealed the presence of bioactive compounds such as gallic acid, caffeic acid, bolinaquinone, dactyloquinone, and others, which are known for their antimicrobial properties. These findings suggest that *D. elegans* has promising antibacterial properties that could be valuable in combating antimicrobial resistance.

## Introduction

One of the major concerns for human health today is antimicrobial resistance (AMR) (Naser et al., 2024). Human activity is the main factor driving its accelerated appearance and spread, especially the improper and excessive use of antibiotics (Lakshmanan et al., 2023) for treating, preventing, or managing human infections. The situation has escalated to a critical level as minor and severe infections become progressively more difficult to cure (Murray et al., 2022). Many of the currently available drugs have not been able to overcome microbial resistance and combat newly emerging diseases (Al-Zereini et al., 2023). AMR is projected to cause 10 million deaths each year by 2050. The declining discovery of new antibiotics and limited pharmaceutical investment exacerbate this worldwide threat (Tang, Millar & Moore, 2023). Without urgent innovation, resistant infections will surpass current treatments (Theuretzbacher et al., 2020).

Natural products, which offer a wide range of bioactive compounds obtained from various natural sources, have played a significant role in the field of drug discovery. These compounds have potential pharmacological characteristics that make them attractive candidates for further investigation (Thomford et al., 2018). Scientists have identified more than 30,000 natural products derived from marine organisms (Lindequist, 2016). Several statistical analyses conducted on marine natural products indicate that sponges are highly prolific in terms of producing isolated biologically active compounds (Hu et al., 2015).

The Red Sea is recognized for its deep waters, which mark it as a hotspot for biodiversity and endemic species (Wooster et al., 2019). The Gulf of Aqaba, which is located in the northeastern part of the Red Sea, is considered to be a deep, narrow, and somewhat isolated water body (Al-Taani et al., 2020). A significant portion of the Gulf falls within the deep-sea category, which describes marine habitats deeper than 200 m (Moore & Squires, 2016) which makes the Gulf different from other similar deep-sea regions. The Gulf reveals an unusual thermal profile, which keeps the water temperatures high even at its deepest points. The high amount of sunlight, along with little circulation of water from the deeper regions, contributes to the warm temperatures. This region also helps in supporting a wide variety of marine life to flourish (Sengupta, Gildor & Ashkenazy, 2024).

Sponges are aquatic invertebrates, belong to the phylum Porifera. Sponges in the ocean are soft-bodied, mostly sedentary animals with a complex mesh of canals and chambers. This structure enables them to pump and filter immense amounts of water, often rich and diverse in bacterial life. These bacteria serve different purposes, including organic matter decomposition, essential nutrient formation, and providing the sponge with protection against harmful bacteria and diseases (Karleskint, Richard & Small, 2013). However, sponges are a source of novel secondary metabolites with intriguing chemical structures, with different biological activities, such as anticancer and antibacterial activities (Hong et al., 2022).

According to previous studies done in the Gulf of Aqaba, several shallow-water sponge species have significant antibacterial properties. For instance, *Grayella cyathophora’s* ethanolic crude extract showed strong antibacterial activity, especially against *Pseudomonas aeruginosa* (El-Damhougy et al., 2017). Furthermore, the distinct conditions of the deep sea, such as its high pressure, low temperature, and lack of light, drive the production of even more unique bioactive compounds, which would support the scientific interest in studying deep-sea sponges for possible biological uses (Back et al., 2021; Skropeta, 2008; Steffen et al., 2022).

This study focuses on identifying three marine sponges collected from the deepest depths of the Jordanian Gulf of Aqaba, a relatively unexplored environment. By investigating the ethanolic extracts of these sponges, the research aims to evaluate their antibacterial potential and uncover the chemical composition of the most active one. The novelty of this work lies in exploring deep-sea sponges from this distinctive habitat, which may have unique bioactive compounds with significant antibacterial potential.

## Materials & Methods

### Sponge collection

**Figure 1:**
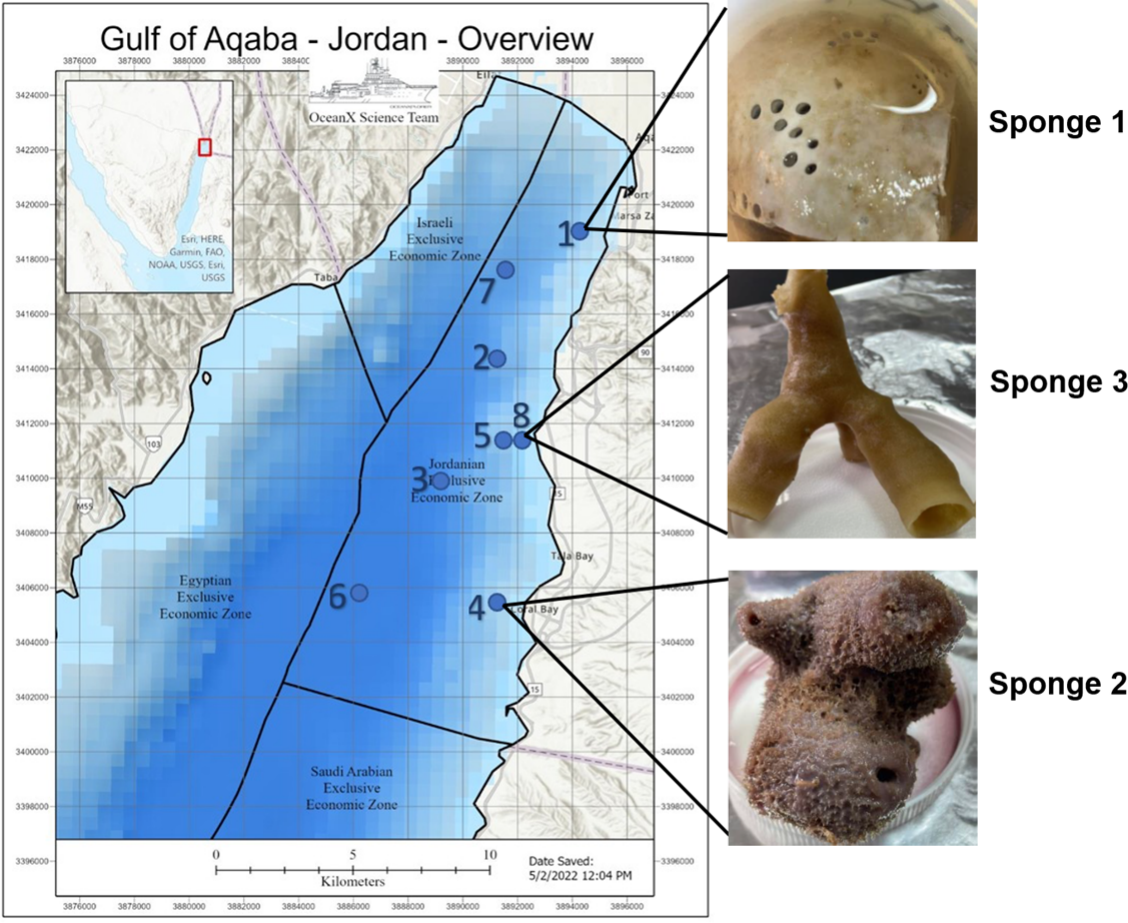
Gulf of Aqaba map and sponge image. Map of the Gulf of Aqaba showing the locations of sample collection and an image of the collected sample. The map was supplied by the OceanX vessel. Numbers 1–8 indicate the ship’s stations during the expedition.

Deep-sea sponge specimens were collected in July 2022 from the Jordanian Gulf of Aqaba using manned submersibles equipped with robotic arms during the OceanXplorer expedition under permit number (9,795), issued by Aqaba special economic zone authority. Ecological parameters were recorded at each collection site. The samples included Sponge 1, collected at a depth of 260 m (temperature: 21.17 °C, salinity: 40.7 PSU, dissolved oxygen: 5.96 mg/L); Sponge 2, collected at 290 m (temperature: 21.1 °C, salinity: 40.7 PSU, dissolved oxygen: 5.92 mg/L); and Sponge 3, collected at 140 m (temperature: 22.5 °C, salinity: 40.66 PSU, dissolved oxygen: 6.21 mg/L). All samples were preserved in 70% ethanol at −4 °C and placed in the Laboratory for Molecular and Microbial Ecology (LaMME), The University of Jordan, Amman. Figure 1 illustrates the location of the sample collection, with an image of the sample.

### Identification of sponge samples

To determine the species of sponge, 25 mg of sponge tissue was divided into tiny fragments. The DNeasy Blood and Tissue Kit from Qiagen (Manchester, UK) was then used to extract the DNA from sponge tissue (Harper et al., 2023). The 28S ribosomal RNA gene served as the molecular marker (Yang et al., 2017), with the primers listed in Table 1. This marker is commonly employed in sponge barcoding for species identification (Timmers et al., 2022).

Gene amplification was performed using the polymerase chain reaction (PCR), veriti™ 96-Well Thermal Cycler (Applied Biosystems, Waltham, MA, USA) with the Enti-link PCR master mix from ELK Biotechnology Co. (Wuhan, China). The PCR was conducted under the following conditions: 3 min of initial denaturation at 94 °C, 35 cycles of denaturation at 94 °C for 30 s, annealing at 56 °C (depending on the gene) for 30 s, 83 s of extension at 72 °C, and a final 10 min of extension at 72 °C. A 1% agarose gel from Cleaver Scientific (Warwickshire, UK) was used to verify the PCR results, which were then examined using a gel documentation system. After that, the PCR products were shipped to Macrogen, South Korea, for sequencing. Finally, we aligned the acquired sequences with sponge species using Molecular Evolutionary Genetics Analysis (MEGA) software by the Center for Evolutionary Medicine and Informatics, Arizona State University, Tempe, AZ, USA. and the Basic Local Alignment Search Tool (BLAST) database from the National Center for Biotechnology Information (NCBI, Bethesda, MD, USA). A phylogenetic tree was constructed using the neighbor-joining method and the aligned 28S rRNA sequences of closely related sponges obtained from the GenBank database collections (Cristianawati et al., 2019).

### Ethanolic extraction

After collecting the sponges, the specimens were immersed in ethanol/water solution (70% v/v) and kept at −20 °C for storage. Ethanol was used to extract both hydrophilic and hydrophobic components in optimum yield (Bashari et al., 2019). The samples were then blended and heated at 55–60 °C for 2 h while being soaked in ethanol, followed by dark resting overnight. The samples were filtered afterward, and the concentrated rinse was rotary evaporated; the paste extract was lyophilized to yield the powder.

**Table 1:** Forward and reverse primer sequences for 28S ribosomal RNA amplification.

Primer	Sequence	Reference
28F63mod (Forward)	5′-ACCCGCTGAAYTTAAGCATATHANTMA-3′	Timmers et al. (2022)
28R1072 (Reverse)	5′-GCTATCCTGAGGGAAACTTCGG-3′	

### Preparation of extract

To make the stock solution, 25 mg of extract powder was dissolved in one ml of dimethyl sulfoxide (DMSO) from Scharlau (Barcelona, Spain) to reach a concentration of 25 mg/ml. This liquid was stored in a Falcon tube after being vortexed and filtered with a 0.45 µm nylon syringe filter. After that, sterile distilled water was used to dilute the stock solution to different concentrations (5, 10, 15, and 20 mg/mL).

### Antimicrobial activity

To cover both Gram-positive and Gram-negative bacteria, six bacterial strains were chosen. Among the Gram-positive bacteria were *Bacillus pumilus* (isolate), *Staphylococcus aureus* American Type Culture Collection (ATCC 29213), and *Staphylococcus epidermidis* (ATCC 51625). Among the Gram-negative bacteria were *Escherichia coli* (ATCC 25922) and *Klebsiella aerogenes* (isolate). Methicillin-resistant *Staphylococcus aureus* (MRSA) (ATCC 1026) is a multidrug-resistant bacterium (MDR) showing resistance to β-lactams, macrolides, fluoroquinolones, and often clindamycin (Chambers & De Leo, 2009). It was subjected to additional testing after the extract showed antibacterial efficacy against *Staphylococcus aureus.*

The antibacterial activity of the three sponge extracts was assessed using the agar well diffusion method. The Muller-Hinton broth (MHB) from Oxoid (Basingstoke, UK) was first mixed with the bacterial strain, and it was then incubated overnight at 37 °C (Magaldi et al., 2004). Following incubation, the density of the culture was modified to satisfy the McFarland turbidity criteria of 0.5 (Salameh et al., 2024).

Using sterile cotton swabs, the test organisms were cultured on Muller-Hinton agar plates, followed by a 10-minute drying period. The agar plates were then prepared with eight mm-diameter wells made with a sterile cork borer. Following that, various concentrations of the sponge extracts (5, 10, 15, and 20 mg/ml) were added to these wells using micropipettes. To allow for pre-diffusion, the culture plates were set on the bench for an hour before being incubated for 24 h at 37 °C (Ibrahim et al., 2022a; Ibrahim et al., 2022b).

Following an 18-hour incubation period, the diameter of the inhibition zones (measured in millimeters) around the wells was used to determine the antibacterial activity (Geetha & Roy, 2013). For all microorganisms, 10 µg gentamycin (Yassine, Ammar & Shabbar, 2013) was utilized as the positive control, and MRSA-specific 30 µg vancomycin (Hannan et al., 2008) was used. Additionally, 80% DMSO was used as the negative control, which was the highest solvent concentration utilized in the extract dilutions. This ensured that any antimicrobial activity observed was due to the extract itself and not the solvent. To reduce the possibility of experimental errors, each experiment was run three times.

### The minimal inhibitory concentration (MIC) and the minimal bactericidal concentration (MBC)

The minimal inhibitory concentration (MIC) procedure, done as in a (Balouiri, Sadiki & Ibnsouda, 2016) protocol with some adjustments, involves the preparation of various extract concentrations using sterile MHB. These concentrations were then added to a 96-well plate in a specific order: for Sponge 2 concentrations: 5, 4, 3, 2, 1.0.5, 0.25, 0.125 mg/ml for all bacterial strains, while Sponge 3 concentrations: 20, 19, 18, 17, 16, 15, 14, 13, 12 mg/ml for *S. aureus* and 15, 14, 13, 12, 11, 10, 9, 8, 7 mg/ml for *S. epidermidis*. The selection of these concentrations depended on the results obtained from the well diffusion agar method. Negative controls included 80% DMSO and sterile MHB, while positive control comprised a bacterial suspension.

The minimal bactericidal concentration (MBC) was established by transferring and evenly spreading the treated culture broth from the wells with concentrations equal to or greater than the MIC onto agar plates. The MBC was defined as the lowest concentration of the fraction needed to entirely eradicate the test microorganism, indicated by no growth observed on the agar plate following incubation at 37 °C for 24 h (Majali et al., 2019).

### Sponge extract chemical composition using LC-MS-MS

Liquid chromatography with tandem mass spectrometry (LC-MS-MS) was performed by Smart Labs Group (Amman, Jordan) using Shimadzu LC (Kyoto, Japan) for the most active sponge extract. It consisted of CBM-20A (control bus module), CTO-30A (column oven), LC-30AD (liquid chromatograph), SIL-30AC (autosampler), and LCMS-8030 (liquid chromatograph mass spectrometer - Triple Quad MS). 50 mg of the sponge extract was extracted with methanol (MeOH). Then, the crude extract was cleaned using a solid phase extraction column, in which the 100% MeOH fraction was retained. A working concentration of 2.5 mg/mL of the crude extract was used. LC-MS spectra consisted of retention time (minutes), accurate molecular ions (>five ppm accuracy), and MS-MS daughter ions. The LC method used for the analysis spanned 15 min with a gradient of 100% H_2_O (with 0.1% formic acid) to 95% acetonitrile (ACN).

### Statistical analysis

All statistical analyses were conducted using GraphPad Prism™ version 10.3.0 (GraphPad Software Inc., San Diego, CA, USA). Two-way ANOVA was used to analyze differences between multiple groups, followed by Tukey’s test for *post-hoc* comparisons. Results were expressed as the mean ± standard error of the mean (SEM), with statistical significance defined as *p* ≤ 0.05.

## Results

### Sponge DNA barcoding

The 28S rRNA gene was successfully amplified from three sponge samples *via* PCR, on agarose gel electrophoresis, yielding bands ranging from 1,000 to 1,300 base pairs (Supplementary 1). Our results confirm high-quality DNA amplification for sequencing.

**Figure 2:**
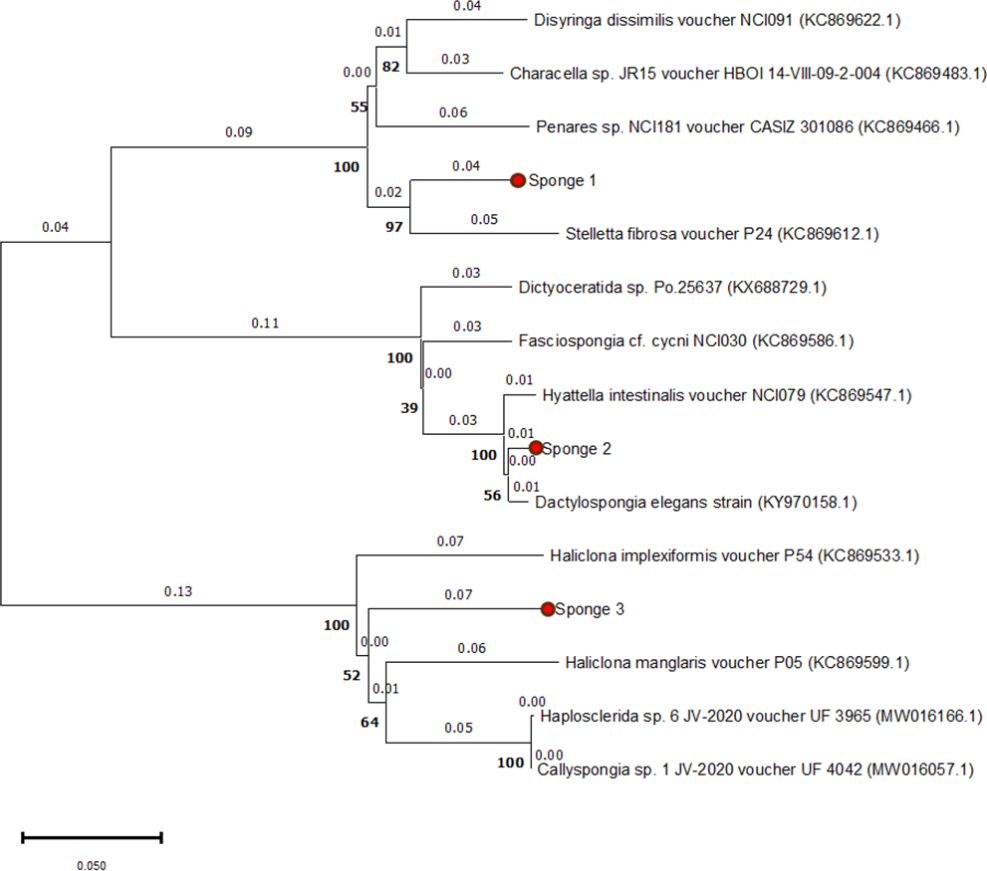
Phylogenetic tree of 28S rRNA sequences from three sponge samples. The phylogenetic tree was constructed using MEGA11 software, with the three sponge samples highlighted in red circles. The tree was generated using the UPGMA method and computed with the Neighbor-Joining algorithm, with 1,000 bootstrap replications (indicated by bold numbers).

The 28S rRNA sequences from the three specimens showed similarities with other Spongillidae species when analyzed using BLAST. The sequences were matched to Spongillidae GenBank sequences that already existed. The following are the findings of the BLAST analysis for the three sponge samples’ 28S rRNA sequence alignment with GenBank. Sponge 1 was determined to be *Stelletta fibrosa*, which is a member of the genus *Stelletta,* class Demospongiae, order Tetractinellida, and family Ancorinidae. The sponge 2 was found to be *Dactylospongia elegans*, which is also a member of the class Demospongiae but belongs to the family Thorectidae, order Dictyoceratida, and genus *Dactylospongia*. Sponge 3 was found to be *Haliclona manglaris*, which is a member of the family Chalinidae, genus *Haliclona*, and order Haplosclerida under the class Demospongiae. The phylogenetic tree constructed using the Neighbor-joining method with 1000 bootstrap support indicates high 28s rRNA sequence similarities with previously mentioned species (Fig. 2).

### Antimicrobial activity

The extracts’ antibacterial activity at various concentrations was evaluated using the agar well diffusion technique, and the diameter of the inhibition zone was measured in millimeters (mm). The results are depicted in Table 2 and Supplementary 2.

**Table 2:** Inhibition zone diameters (mm) for different bacterial strains treated with sponge ethanolic extracts in different concentrations. Data are expressed as mean ± standard error of the mean (SEM) based on three independent replicates (*n* = 3), positive control for all bacteria: Gentamycin (10 µg), MRSA-specific positive control: vancomycin (30 µg), and negative control: 80% DMSO.

		**Mean of the inhibition zone diameter (mm) (Mean** ± SEM)					
	**Bacterial strains**	*E. coli*	*S. aureus*	*K. aerogenes*	*B. pumilus*	*S. epidermidis*	MRSA
*D. elegans* (sponge 2)	5 mg/ml	0.0 ± 0.0	8.6 ± 4.4	0.0 ± 0.0	6 ± 2.5	12.6 ± 2.3	11.6 ± 1.6
	10 mg/ml	0.0 ± 0.0	16.3 ± 2.1	0.0 ± 0.0	11.3 ± 0.6	15.6 ± 2.4	13.6 ± 1.2
	15 mg/ml	0.0 ± 0.0	18 ± 2.0	0.0 ± 0.0	14.6 ± 1.7	19.3 ± 1.2	14.6 ± 0.6
	20 mg/ml	0.0 ± 0.0	19.6 ± 1.3	0.0 ± 0.0	18 ± 1.1	21 ± 0.5	15.3 ± 0.8
	Positive control	14 ± 0.57	17.6 ± 2.9	12.3 ± 2.1	21.6 ± 0.6	21.3 ± 1.6	21.6 ± 0.3
	Negative control	0.0 ± 0.0	0.0 ± 0.0	0.0 ± 0.0	0.0 ± 0.0	0.0 ± 0.0	0.0 ± 0.0
*H. manglaris* (sponge 3)	5 mg/ml	0.0 ± 0.0	0.0 ± 0.0	0.0 ± 0.0	0.0 ± 0.0	0.0 ± 0.0	0.0 ± 0.0
	10 mg/ml	0.0 ± 0.0	0.0 ± 0.0	0.0 ± 0.0	0.0 ± 0.0	0.0 ± 0.0	0.0 ± 0.0
	15 mg/ml	0.0 ± 0.0	0.0 ± 0.0	0.0 ± 0.0	0.0 ± 0.0	12.0 ± 0.0	0.0 ± 0.0
	20 mg/ml	0.0 ± 0.0	10.6 ± 0.3	0.0 ± 0.0	0.0 ± 0.0	13.0 ± 0.0	0.0 ± 0.0
	Positive control	14.3 ± 0.8	17.1 ± 1.0	17.6 ± 1.7	18.6 ± 1.8	20.6 ± 0.8	21.6 ± 0.3
	Negative control	0.0 ± 0.0	0.0 ± 0.0	0.0 ± 0.0	0.0 ± 0.0	0.0 ± 0.0	0.0 ± 0.0

*S. fibrosa* (Sponge 1) demonstrated no positive results against the selected bacteria. In contrast, *D*. *elegans* (Sponge 2) exhibited significant activity against Gram-positive bacteria (S*. aureus, B. pumilus, S. epidermidis*, and MRSA). However, *H. manglaris* (Sponge 3) showed only modest activity against two bacterial strains (*S. aureus* and *S. epidermidis*).

Positive controls, gentamycin (10 µg) and vancomycin (30 µg), showed clear inhibitory zones against all tested microorganisms, validating the technique. In contrast, the negative control (80% DMSO) exhibited no inhibition, Table 2, showing that the observed antibacterial activity was primarily due to sponge extracts.

**Table 3:** Compounds detected in the ethanol extract of *D. elegans* (Sponge 2).

	Compounds	Molecular formula	Molecular weight	%	RT
1	Limonene	C_10_H_16_	136.23 g/mol	1.1	1
2	Ocimene	C_10_H_16_	136.23 g/mol	0.2	1.1
3	Indole-3- carbaldehyde	C_9_H_7_NO	145.16 g/mol	2.2	1.2
4	Gallic acid	C_7_H_6_O_5_	170.12 g/mol	10.1	1.3
5	Caffeic acid	C_9_H_8_O_4_	180.16 g/mol	8	1.4
6	Hyatellaquinone	C_12_H_14_O_4_	222.24 g/mol	3.2	1.5
7	Linoleic acid	C_18_H_32_O_2_	280.4 g/mol	6.6	1.6
8	Catechin	C_15_H_14_O_6_	290.27 g/mol	1	1.68
9	Clathric acid	C_20_H_30_O_2_	302.5 g/mol	0.5	1.7
10	Chromazonarol	C_21_H_30_O_2_	314.5 g/mol	5.1	1.8
11	Smenospongine	C_21_H_29_NO_3_	343.5 g/mol	1.5	1.9
12	Dysideamine	C_21_H_29_NO_3_	343.5 g/mol	2	2
13	Chlorogenic acid	C_16_H_18_O_9_	354.31 g/mol	0.9	2.1
14	Dactyloquinone	C_22_H_28_O_4_	356.5 g/mol	7.1	2.25
15	Smenospongimine	C_22_H_31_NO_3_	357.5 g/mol	1.2	2.3
16	Ilimaquinone	C_22_H_30_O_4_	358.5 g/mol	4.3	2.4
17	Isospongiaquinone	C_22_H_30_O_4_	358.5 g/mol	2.1	2.6
18	Bolinaquinone	C_22_H_30_O_4_	358.5 g/mol	7.4	2.8
19	Mamanuthaquinone	C_22_H_30_O_4_	358.5 g/mol	6.1	2.9
20	Cyclospongiaquinone	C_22_H_30_O_4_	358.5 g/mol	3.1	3.1
21	Dactyltronic acid	C_21_H_30_O_5_	362.5 g/mol	0.6	3.15
22	Dictyoceratin A	C_23_H_32_O_4_	372.5 g/mol	0.3	3.3
23	Pelorol	C_23_H_32_O_4_	372.5 g/mol	5.1	3.5
24	Luffariellolide	C_25_H_38_O_3_	386.6 g/mol	0.8	3.65
25	Ergosterol	C_28_H_44_O	396.6 g/mol	4.1	3.8
26	*Manoalide*	C_25_H_36_O_5_	416.5 g/mol	6	4
27	Motualevic acid	C_16_H_23_Br_2_NO_2_	421.2 g/mol	1.3	4.2
28	Lectin	C_26_H_28_O_6_	436.5 g/mol	3	4.4
29	Nakijiquinone D	C_25_H_35_NO_6_	445.5 g/mol	1.6	4.6
30	Stelletin	C_30_H_38_O_4_	462.6 g/mol	1.7	4.9
31	Manzamine A	C_36_H_44_N_4_O	548.8 g/mol	0.3	5.2
32	Rutin	C_27_H_30_O_16_	610.5 g/mol	0.5	5.4
33	Popolohuanone	C_42_H_57_NO_3_	623.9 g/mol	1	5.6

**Figure 3:**
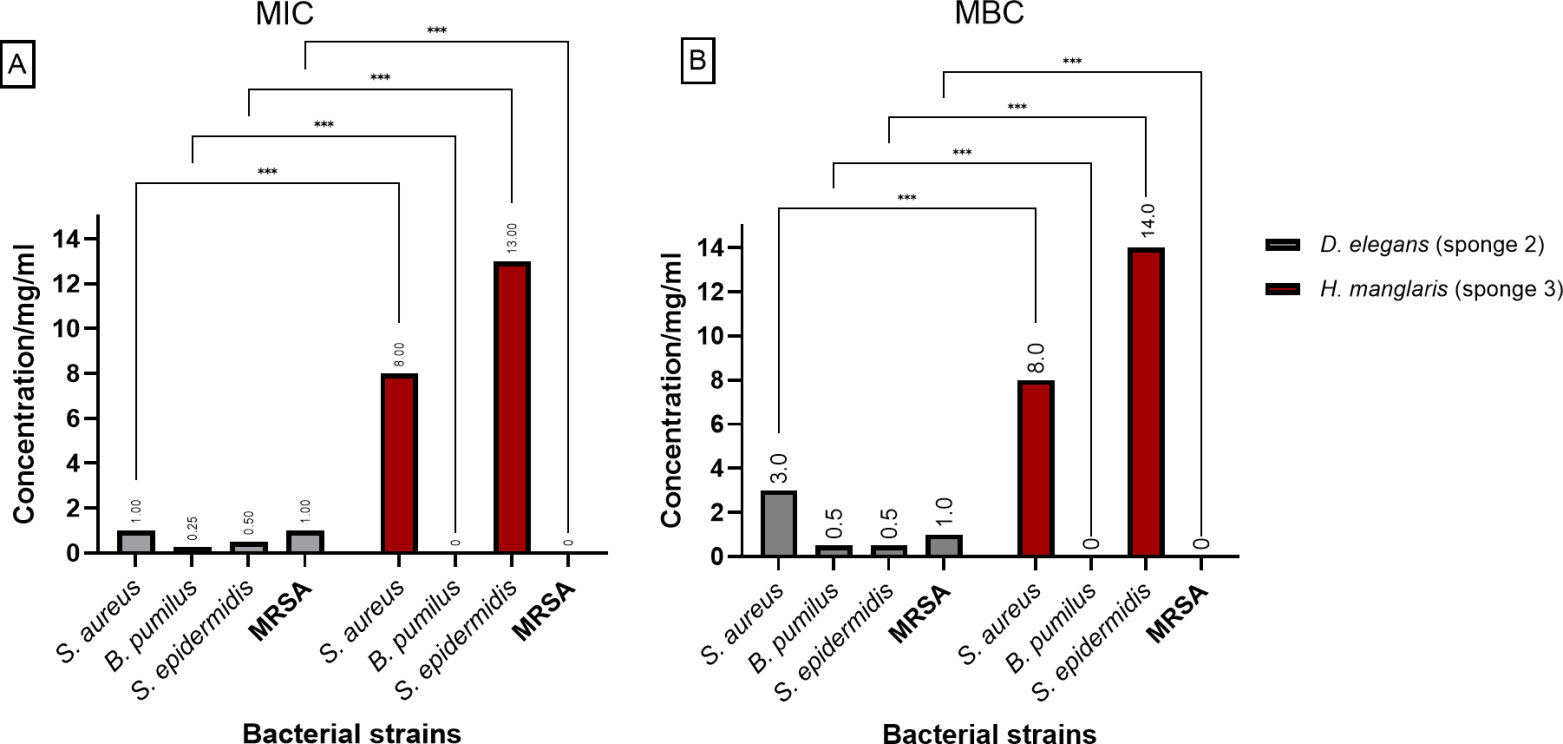
MIC and MBC of *D. elegans* (sponge 2) and *H. manglaris* (sponge 3) ethanolic extracts: (A) MIC and (B) MBC for the ethanolic extracts of *D. elegans* (Sponge 2) against *S. aureus, B. pumilus, S. epidermidis,* and MRSA, as well as *H. manglaris* (Sponge 3) against *S. aureus* and * S. epidermidis*. Statistical analysis revealed significant differences between the two sponge extracts, with *p* values indicated as follows: *p* = 0.033 (*), *p* = 0.002 (**), and *p* < 0.001 (***). SEM values were zero in all cases; therefore, error bars are not displayed.

Figure 3 presents the (MIC) and (MBC) values for the extracts from *D. elegans* (Sponge 2) and *H. manglaris* (Sponge 3), both of which exhibited antibacterial activity against the tested bacteria. Statistical analysis revealed significant differences (*p* < 0.05) between the two sponge extracts for each bacterial strain. At lower concentrations, *D. elegans* (Sponge 2) yielded promising results, especially against *B. pumilus*, with a MIC of 0.125 mg/mL and an MBC of 0.5 mg/mL. In contrast, *H. manglaris* (Sponge 3) showed notable effectiveness against *S. aureus*, with both its MIC and MBC values reaching eight mg/mL.

### LC-MS-MS

Through LC-MS-MS analysis (Supplementary 3), the ethanol extract of *D. elegans* (sponge 2) was characterized as in Table 3. The prominent compounds identified were a high proportion (over 7%) of gallic acid, caffeic acid, dactyloquinone, and bolinaquinone. Other significant metabolites, including indole-3-carbaldehyde, hyatellaquinone, linoleic acid, chromazonarol, ilimaquinone, isospongiaquinone, mamanuthaquinone, cyclospongiaquinone, pelorol, ergosterol, manoalide, and lectin, are also present at more than 2% in the extract.

## Discussion

The marine environment is incredibly rich in a diverse array of species, offering a vast reservoir of biometabolites. Many of these biometabolites possess unique chemical structures that are not found in terrestrial sources. This study demonstrated that *D. elegans* ethanolic extract exhibited promising selective antibacterial activity against gram-positive bacteria *S. aureus, S. epidermidis, B. pumilus,* and MRSA, and *H. manglaris* ethanolic extract exhibited weak antibacterial activity against *S. aureus* and *S. epidermidis*.

The marine sponge *Haliclona manglaris* has antibacterial properties, especially against Gram-positive bacteria (Nazemi et al., 2014). This action stems from bioactive substances produced by the sponge itself, such as lectins (Carneiro et al., 2015), which shows promising activity against biofilm formation (Andrade et al., 2023), as well as from compounds produced by its symbiotic microbes (Gupta, 2019).

*Haliclona* sp. from the Persian Gulf was tested against gram-positive and gram-negative bacteria in aqueous, diethyl ether, and methanol extracts. With MIC = 5 mg/ml, the diethyl ether extract exhibited the highest activity level, especially against *B. subtilis* and *S. aureus* (Nazemi et al., 2014).

Dactylospongia sponges, notably *D. elegans*, are well-known for producing a vast spectrum of metabolites with potent bioactivities. The majority of these metabolites are made up of sesquiterpenes, which include hydroquinones, quinones, and tetronic acids, as well as a few sesterterpenes, sterols, and pregnanes. These compounds have shown substantial bioactivities, including anticancer, cytotoxic, antibacterial, and anti-inflammatory properties (Ibrahim et al., 2022a; Ibrahim et al., 2022b).

The LC-MS-MS analysis of the *D. elegans* ethanolic extract shows it contains high concentration of novel bioactive metabolites, particularly since it was collected from a unique marine environment with stable warm temperatures (above 20 °C) and high oxygen levels (∼six mg/L), so the conditions that may have impacted the production of metabolites. The antibacterial activity of these metabolites is deeply linked to their unique ecological conditions, acting as a defense against biofouling and microbial hazards, with their particular chemical arsenal shaped by environmental stressors like nutrient levels and temperature (Faulkner, 1984; Tähtinen et al., 2018).

Since phenolic chemicals like gallic acid, caffeic acid, chromazonarol, and pelorol are often found in terrestrial plants, their discovery in a marine-derived extract is especially remarkable. This compound shows strong antibacterial activity, especially against Gram-positive bacteria like *Staphylococcus aureus* (Pinho et al., 2014). This result is in line with earlier research that has documented the potency of these molecules against related bacterial strains.

Gallic acid, in particular, is highly effective even at low dosages, primarily by rupturing the structural integrity of the bacterial cell membrane, which eventually results in bacterial cell death (Luís et al., 2014). Furthermore, other research has shown its ability to inhibit bacterial growth by changing bacterial metabolism and lowering the formation of biofilms (Keyvani-Ghamsari, Rahimi & Khorsandi, 2023). Caffeic acid exhibits strong antibacterial properties individually and with other drugs (Aijaz et al., 2022). As a comprehensive review conducted by Khan et al. (2021), caffeic acid has antibacterial activity through several mechanisms, such as bacterial cell membrane disruption, inhibiting essential enzymes, and interfering with microbial pathogenicity. Strong activity of caffeic acid against *S. aureus* with MIC and MBC (0.625 and 1.25 mg/mL) was recorded, most likely as a result of hyper-acidification that caused bacterial cell disruption. Additionally, it was effective against *S. epidermidis* (MIC and MBC: 0.625 mg/mL), presumably by donating protons that increase the internal acidity of the cell (Pinho et al., 2014). Gallic acid and caffeic acid have not been previously isolated from this species, as reported by Ibrahim et al. (2022a); Ibrahim et al. (2022b) but have been isolated previously from sponge-associated bacteria from the sponge *Hyrtios erectus* (Ma et al., 2019b). Pelorol also showed moderate to strong antimicrobial activity, specifically against *S. aureus* and *E. faecalis,* with MICs of 3.125 and 12.5 µM, respectively (Ebada et al., 2017).

Bolinaquinone, dactyloquinone, hyatellaquinone, ilimaquinone, isospongiaquinone, mamanuthaquinone, and cyclospongiaquinone are marine sesquiterpene quinones. Among them, hyatellaquinone showed comparatively high action against MRSA and *S. aureus* (Ma et al., 2019a). Likewise, ilimaquinone shows antibacterial properties against *Streptococcus pyogenes* and *S. aureus* (Chen et al., 2022), but isospongiaquinone demonstrated specific activity against *S. aureus* only (Ebada et al., 2017). Additionally, quinone derivatives have been proven to target essential bacterial proteins, such as LptA and Top IV, suggesting a dual-target mechanism of antibacterial action (Yang et al., 2019). The variety in structure among these compounds, especially in their quinone moieties and terpenoid skeletons, may contribute to their effectiveness and lower the threat of resistance development (Emami, Shafiei & Foroumadi, 2005).

Linoleic acid has activity against *S. aureus* by rupturing bacterial membranes with MIC values as low as 0.01 mg/mL (Dilika, Bremner & Meyer, 2000). Indole-3-carbaldehyde has been recorded to inhibit bacterial growth and biofilm formation, with MIC values of 100 and 150 µg/mL against *S. aureus* and *B. subtilis*, respectively, suggesting it interferes with cell signaling and virulence factors (Rattanaphan et al., 2020). Lectins are sugar-binding proteins that effectively inhibit bacterial adhesion and biofilm formation (Hasan et al., 2023). Ergosterol has shown antibacterial effects, likely through disrupting the membrane integrity and interfering with bacterial metabolism (Andrade et al., 2019). However, to the best of our knowledge, no studies have reported the antibacterial activity of bolinaquinone, dactyloquinone, mamanuthaquinone, cyclospongiaquinone, chromazonarol, and manoalide, so further research is needed to assess their potential as antimicrobial agents.

Moreover, apart from individual compounds’ reported activity in literature, the antibacterial activity of the extract may have risen due to synergistic activity between two or more compounds present in the extract. These could be of high or low abundance within the extract. Further studies in terms of purifying the extract and isolating each compound to analyze it alone, MS analysis, and antibacterial activity, would be worth investigating. Also, combining isolated compounds to study any possible synergistic antibacterial activity would be worth studying

In conclusion, this study opens the door for further research into novel antimicrobial drugs by demonstrating the antibacterial activity of the deep *D. elegans* ethanolic extract and highlighting its bioactive components discovered through LC-MS/MS analysis. Although bolinaquinone, dactyloquinones, and other molecules have mostly been examined for their anti-inflammatory activities and cytotoxicity, their potential antibacterial action remains unexplored. More research is necessary to determine their antibacterial properties and to investigate if they have synergistic interactions with gallic acid, caffeic acid, and others. Such investigations could provide important information about their combined therapeutic potential as novel antibacterial agents.

##  Supplemental Information

10.7717/peerj.19735/supp-1 Supplemental Information 1 Agarose Gel Electrophoresis of 28S rRNA PCR Products from Three Sponge Samples The PCR amplification results of the 28S rRNA gene for three sponge samples. Each well was loaded with 3 *μ*L of PCR product. Lane 1 contains the negative control, Lane 2 represents Sponge 1, Lane 3 represents Sponge 2, Lane 4 represents Sponge 3, and Lane 5 contains the 1 Kb molecular ladder for size reference.

10.7717/peerj.19735/supp-2 Supplemental Information 2 Agar well diffusion result The results of the agar well diffusion assay for antibacterial activity against: *E. coli*, *K. aerugen, S. aureus, B. pumilus, S. epidermidis*, and MRSA. (A) *S. fibrosa* (sponge 1), (B) *D. elegans* (sponge 2), (C) *H. manglaris* (sponge 3). With concentrations (5, 10, 15, 20 mg/ml). Gentamycin (10*μ*g): Positive control for all bacteria, Vancomycin (30*μ*g): MRSA-specific positive control, and 80%DMSO: Negative control.

10.7717/peerj.19735/supp-3 Supplemental Information 3 Chromatogram of *D. elegans* (sponge 2) ethanolic extract

10.7717/peerj.19735/supp-4 Supplemental Information 4 SEM analysis of sponge 2- well diffusion result

10.7717/peerj.19735/supp-5 Supplemental Information 5 SEM analysis of sponge 3- well diffusion

10.7717/peerj.19735/supp-6 Supplemental Information 6 Raw data 2-way ANOVA of MIC

10.7717/peerj.19735/supp-7 Supplemental Information 7 Raw data 2-way ANOVA of MBC

## Additional Information and Declarations

### Field Study Permissions

The following information was supplied relating to field study approvals (i.e., approving body and any reference numbers): Aqaba special economic zone authority.

### DNA Deposition

The following information was supplied regarding the deposition of DNA sequences: The *Stelletta fibrosa* are available at GenBank: KC869612.1. The *Dactylospongia elegans* are available at GenBank: KY970158.1. The *Haliclona manglaris* are available at GenBank: KC869599.1

### Data Availability

The following information was supplied regarding data availability: The raw data is available in the Supplementary Files.
