# Supplementary figures and images for: Evaluating the antibacterial properties of deep-sea sponges Dactylospongia elegants, Stelletta fibrosa, and Haliclona manglaris from the Jordanian Gulf of Aqaba
Source: PeerJ. 2025 Jul 31;13:e19735. doi: 10.7717/peerj.19735 (PMC12318501; doi:10.7717/peerj.19735)

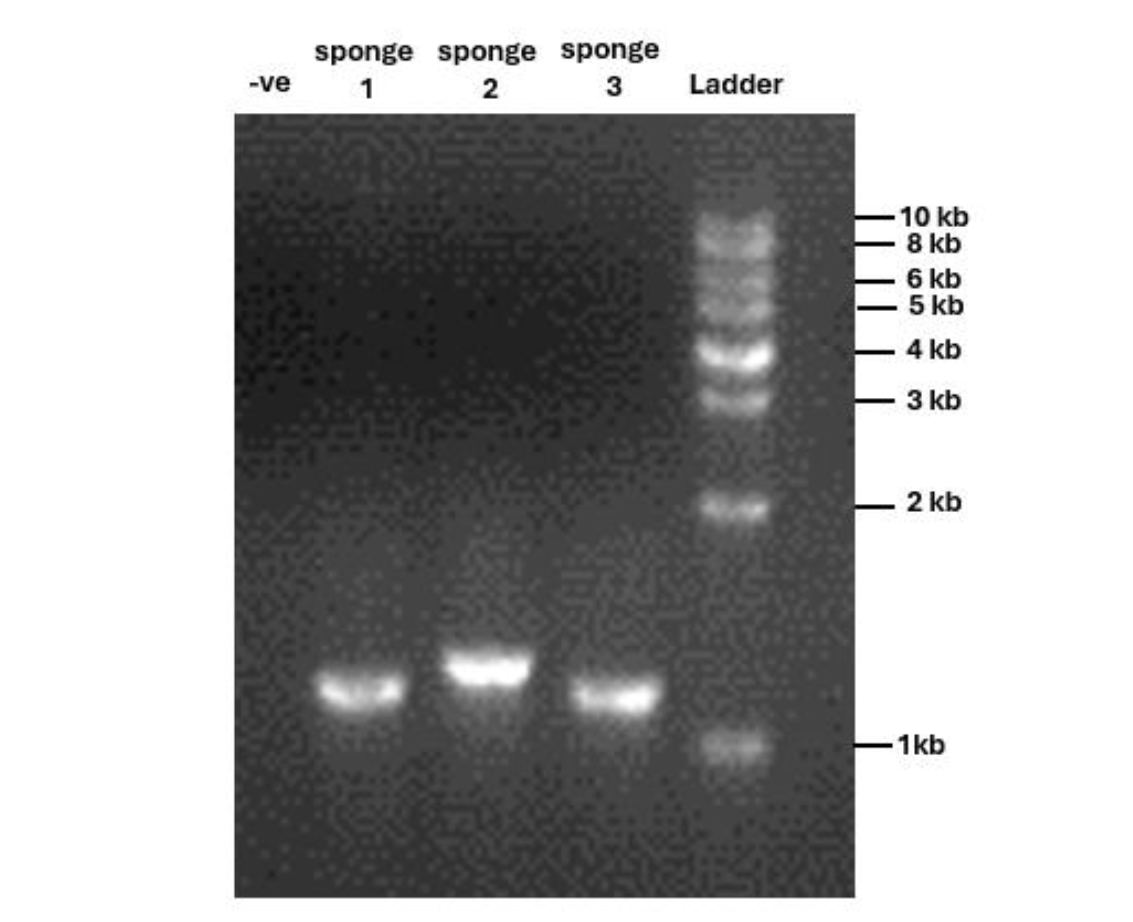

Supplement: Supplemental Information 1 — The PCR amplification results of the 28S rRNA gene for three sponge samples. Each well was loaded with 3 μL of PCR product. Lane 1 contains the negative control, Lane 2 represents Sponge 1, Lane 3 represents Sponge 2, Lane 4 represents Sponge 3, and Lane 5 contains the 1 Kb molecular ladder for size reference. [file peerj-13-19735-s001.png]

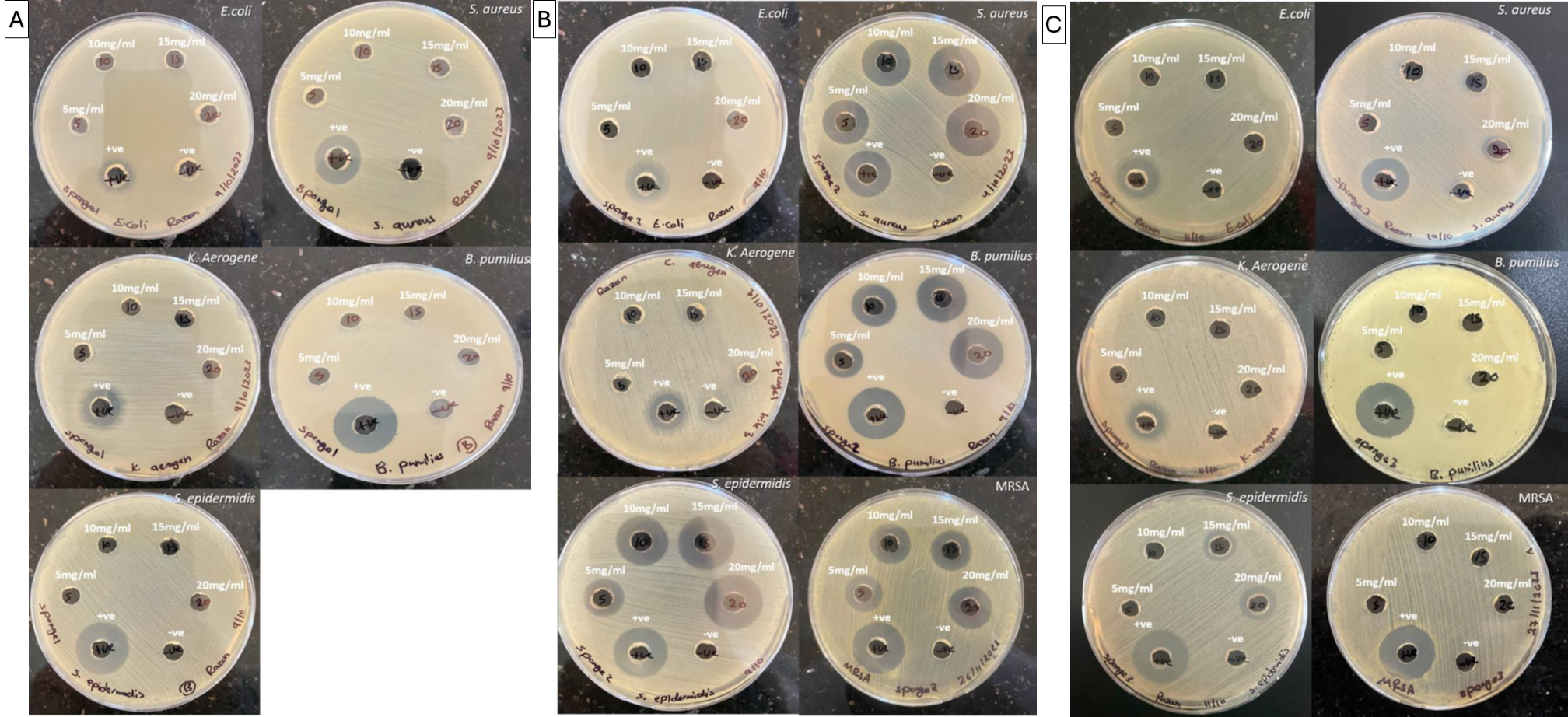

Supplement: Supplemental Information 2 — The results of the agar well diffusion assay for antibacterial activity against: E. coli, K. aerugen, S. aureus, B. pumilus, S. epidermidis, and MRSA. (A) S. fibrosa (sponge 1), (B) D. elegans (sponge 2), (C) H. manglaris (sponge 3). With concentrations (5, 10, 15, 20 mg/ml). Gentamycin (10μg): Positive control for all bacteria, Vancomycin (30μg): MRSA-specific positive control, and 80%DMSO: Negative control. [file peerj-13-19735-s002.png]

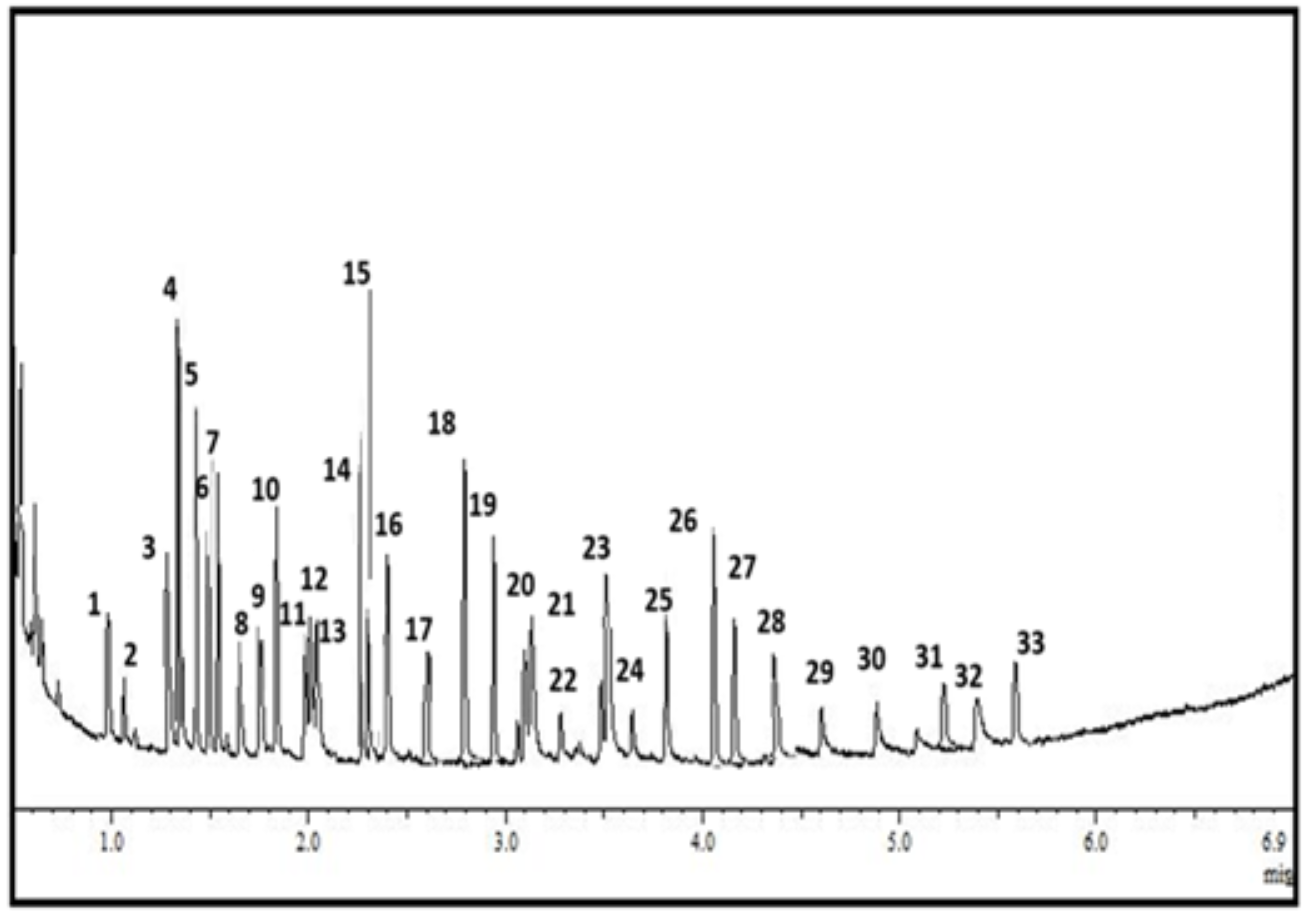

Supplement: Supplemental Information 3 [file peerj-13-19735-s003.png]
